# High-Throughput Screening of a Functional Human CXCL12-CXCR4 Signaling Axis in a Genetically Modified *S. cerevisiae*: Discovery of a Novel Up-Regulator of CXCR4 Activity

**DOI:** 10.3389/fmolb.2020.00164

**Published:** 2020-07-16

**Authors:** James W. Murphy, Deepa Rajasekaran, Janie Merkel, Erin Skeens, Camille Keeler, Michael E. Hodsdon, George P. Lisi, Elias Lolis

**Affiliations:** ^1^Department of Pharmacology, Yale School of Medicine, Yale University, New Haven, CT, United States; ^2^Yale Center for Molecular Discovery, Yale School of Medicine, Yale University, New Haven, CT, United States; ^3^Department of Molecular Biology, Cell Biology and Biochemistry, Brown University, Providence, RI, United States; ^4^Department of Laboratory Medicine, Yale School of Medicine, Yale University, New Haven, CT, United States; ^5^Yale Cancer Center, New Haven, CT, United States

**Keywords:** chemokine, G protein-coupled receptor, CXCL12, CXCR4, high-throughput screening, fosfosal, NMR

## Abstract

CXCL12 activates CXCR4 and is involved in embryogenesis, hematopoiesis, and angiogenesis. It has pathological roles in HIV-1, WHIM disease, cancer, and autoimmune diseases. An antagonist, AMD3100, is used for the release of CD34+ hematopoietic stem cells from the bone marrow for autologous transplantation for lymphoma or multiple myeloma patients. Adverse effects are tolerated due to its short-term treatment, but AMD3100 is cardiotoxic in clinical studies for HIV-1. In an effort to determine whether *Saccharomyces cerevisiae* expressing a functional human CXCR4 could be used as a platform for identifying a ligand from a library of less ∼1,000 compounds, a high-throughput screening was developed. We report that 2-carboxyphenyl phosphate (fosfosal) up-regulates CXCR4 activation only in the presence of CXCL12. This is the first identification of a compound that increases CXCR4 activity by any mechanism. We mapped the fosfosal binding site on CXCL12, described its mechanism of action, and studied its chemical components, salicylate and phosphate, to conclude that they synergize to achieve the functional effect.

## Introduction

CXCR4 is a GPCR with physiological roles in cellular homeostasis and a variety of immune response functions, and is activated by the chemokine agonist CXCL12. The agonist exists as a monomer (Crump et al., 1997), dimer (Dealwis et al., 1998), and a decamer (Murphy et al., 2010). Although the monomeric form activates the receptor (Kofuku et al., 2009; Qin et al., 2015), interactions of the various oligomeric states with glycoaminoglycans play a role in establishing a concentration gradient in tissues that allows immune cells expressing CXCR4 to migrate in response to increased CXCL12 concentrations (Fernandez and Lolis, 2002; Handel et al., 2005; Allen et al., 2007). Due to the importance of the monomer in activating the receptor and the oligomers in establishing this concentration gradient, most biophysical studies have investigated this monomer–dimer equilibrium, including factors that alter the equilibrium such as pH, phosphate or sulfate ions, and heparin (Veldkamp et al., 2005; Murphy et al., 2007).

In homeostasis, the monomer and higher order oligomers regulate the release of CD34+ hematopoietic stem cells from the bone marrow (Aiuti et al., 1997). The drug AMD3100 (Plerixafor), a CXCR4 antagonist, was approved as a second-line treatment for patients with multiple myeloma or non-Hodgkin’s lymphoma (De Clercq, 2009); however in a clinical trial as a HIV-1 therapeutic [CXCR4 is one of two co-receptors for HIV-1 (Feng et al., 1996)], AMD3100 was withdrawn from Phase 1 within 30 days due to premature ventricular contractions (Hendrix et al., 2004). Other studies are consistent with CXCL12-CXCR4 cardioprotection during pre- and post-myocardial ischemia, which is prevented by AMD3100 or a CXCR4 inducible knockout in cardiac myocytes (Hu et al., 2007; Huang et al., 2011; Dong et al., 2012; LaRocca et al., 2019). However, the effects of CXCL12-CXCR4 on the cardiovascular system are complicated by varied cell types and cardiac events, indicating more studies are necessary to define mechanisms where activation or antagonism of CXCR4 are beneficial (Bernhagen et al., 2007; Zernecke et al., 2008; Doring et al., 2017, 2019).

AMD3100 antagonism of CXCR4 would be a viable therapeutic approach for other diseases if a strategy to prevent its toxicity is identified. Some of these clinical indications for CXCR4 antagonism include infection by T-tropic strains of HIV which immediately precede AIDS (Feng et al., 1996; Doranz et al., 1997; Donzella et al., 1998), gain-of-function mutations in the orphan disease WHIM syndrome (Walters et al., 2010), and over 20 metastatic cancers (Chatterjee et al., 2014). Short-term treatment of AMD3100 in clinical settings successfully ablated the symptoms of WHIM although it has not been studied long term due to its toxicities (Walters et al., 2010; Dale et al., 2011; McDermott et al., 2011, 2019; Peled et al., 2012). Strategies or compounds that cause biased agonism/antagonism or increased CXCR4 activity may have significant research interest and provide a foundation for possible therapeutics.

Interestingly, AMD3100 functions as a partial agonist for a constitutively active mutant (CAM) of CXCR4, suggesting the feasibility of identifying small molecule agonists for the wild-type form of the receptor (Zhang et al., 2002; Rosenberg et al., 2019). We previously used a genetically manipulated strain of *Saccharomyces cerevisiae* expressing a functional human CXCR4 to identify two allosteric agonists from a cDNA library coding for ∼160,000 peptides derived from CXCL12 (Sachpatzidis et al., 2003). In the work reported here, we were interested in determining whether this *S. cerevisiae* strain could be used in a proof-of-principle experiment to identify a small molecule that antagonizes CXCR4 signaling from a small library of 960 bioactive compounds. Surprisingly, we found a compound, 2-carboxyphenyl phosphate (fosfosal), that increased CXCR4 activity but only in the presence of CXCL12. Fosfosal is a pro-drug for salicylate but has better gastric tolerance compared to acetylsalicylic acid and is used in Spain as a nonsteroidal anti-inflammatory drug (NSAID) (Rafanell et al., 1980). The effect of fosfosal in *S. cerevisiae* expressing CXCR4 in the presence of 200 nM CXCL12 increased signaling at the highest levels of fosfosal. Nuclear magnetic resonance (NMR) was used to determine the affinity of fosfosal for CXCL12, map the site of interaction, and, together with size exclusion chromatography with multi-angle light scattering (SEC-MALS) and molecular docking, determine the mechanism of action. We also evaluated the chemical components of fosfosal, salicylic acid and phosphate, to determine how these chemical groups contribute to fosfosal binding to CXCL12.

## Materials and Methods

### High-Throughput (HTS) Optimization of *S. cerevisiae* Expressing a Functional Human CXCR4

All chemicals and reagents were used as received. The Microsource (Microsource Discovery System, Inc., Gaylordsville, CT, United States) small molecule library, consisting of 960 compounds with known biological activity, was used for screening. High-throughput screening (HTS) assays were optimized to detect antagonists of CXCR4 using the agonist CXCL12 and antagonist AMD3100 as controls.

The genetically modified strain of *S. cerevisiae* (strain CY12946) used in the HTS was transformed with two plasmids (Sachpatzidis et al., 2003), pCXCR4 and pFus1-βgal, constitutively expressed human CXCR4 and the Fus1-dependent promoter for β-galactosidase (β-gal), respectively. The Fus promoter produced β-gal only when CXCR4 was activated by cell signaling. Other genetic changes to this *S. cerevisiae* strain that make this system appropriate for HTS were: (1) the replacement of the yeast GPCR Ste3 of the pheromone response pathway with CXCR4, thereby eliminating all other GPCRs for selectivity, (2) the replacement of five C-terminal residues of yeast Gα (Gpa1) with those from human Gα_i2_ to allow coupling with CXCR4, (3) the deletion of *Ste14* (coding for a methyltransferase) to dampen background activity of the pheromone response pathway, (4) the deletion of *Sst2* that codes for a RGS, a negative regulator of G-protein signaling to facilitate a more robust MAP kinase signaling response (Dohlman et al., 1996), (5) the deletion of *Far1*, which normally leads to cell cycle arrest, to promote a long-lived signaling response, (6) the addition of the *Trp1* gene as a selective marker for *lacZ*, and (7) the transcription of *lacZ* upon MAP kinase signaling, allowing β-galactosidase to provide a quantitative measure of CXCR4 activation.

Based on high-throughput optimization, *S. cerevisiae* strain CY12946 previously transformed with pCXCR4 and pFus1-βgal was grown overnight to saturation (OD_600_ = 1.5) in nutritional selective dropout media and diluted to 55,000 cells/10 μL. Using three Corning #3574 non-binding white 384-well plates for the screen, 10 μL of complete minimal (CM) dropout media was distributed with a bulk reagent dispenser (Thermo Scientific Multidrop) into all wells. A positive control (100% antagonism) consisted of 10 μL of cells treated with 10 μM AMD3100 with 200 nM CXCL12 added to columns 1 and 2. CXCL12-treated cells were added to columns 3–22 followed by compounds from the library using a pin-tool device. Cells treated with only 200 nM CXCL12 were added to columns 23–24 as the negative control (0% antagonism). Plates were sealed, incubated with shaking at 30°C for 4 h, and treated with 20 μL of Beta-Glo (Promega, converts β-galactosidase activity to luminescence) for 25 min at 25°C. Luminescence measurements were made using an Envision plate reader. Signal to background for the assay was 28:1 and calculated by the mean luminescence of CXCL12 stimulated cells divided by mean luminescence of cells treated with AMD3100 followed by CXCL12. The Z’ value for the assay was >0.8 (Zhang et al., 1999). Wells containing potential antagonists were to be assessed as % inhibition with 0% and 100% representing the mean of columns 23–24 and 1–2, respectively.

### Protein Expression and Purification of CXCL12 and ^15^N-CXCL12

CXC chemokine ligand 12 was expressed and purified as previously described (Murphy et al., 2007). Briefly, a modified human CXCL12α cDNA clone without the secretion sequence, but with a codon for an initiating Met and a deleted codon for the C-terminal Lys68, was cloned into the *Nde*I and *Xho*I restriction sites of the pET-22b expression vector and transformed into the *Escherichia coli* BL21(DE3) strain. Luria–Bertani media (1.5 L) with 100 μg/ml ampicillin was inoculated with BL21(DE3), grown to OD_600_ of 0.6, and induced with a final concentration of 1.0 mM isopropyl 1-thio-β-d-galactopyranoside. After expressing CXCL12 for an additional 4 h at 37°C, cells were harvested by centrifugation at 5,000 × *g* for 10 min. Cells were resuspended in phosphate-buffered saline at pH 7.4 and 1% Triton X-100, lysed using a French Press, and centrifuged for 30 min at 30,000 × *g*. CXCL12 was found in the inclusion bodies, which were washed three times in a buffer containing 100 mM Tris⋅HCl, pH 7.0, 5 mM EDTA, 5 mM DTT, 2 M urea, 2% Triton X-100 and once with wash consisting of 100 mM Tris⋅HCl pH 7.0, 5 mM EDTA, and 5 mM DTT. The inclusion bodies were solubilized with 6 M guanidine HCl, diluted 100-fold into a refolding buffer consisting of 100 mM Tris⋅HCl, pH 8.0, 5 mM EDTA, 0.2 mM oxidized glutathione, and 1 mM reduced glutathione, and stirred at 4°C overnight. Precipitated material was removed, and the refolded protein was applied to an SP Sepharose column and eluted by a NaCl gradient. CXCL12 fractions were combined and further purified by RP-HPLC. Fractions containing CXCL12 were concentrated and lyophilized. Pure CXCL12 was resuspended in sterile H_2_O (pH 7.0) with 0.1 mM NaN_3_ and a protease inhibitor cocktail prior to use. For NMR studies, isotopically labeled ^15^N-CXCL12 was expressed in M9 minimal media containing 1 g/L of ^15^NH_4_Cl (98%-^15^N), IPTG-induced, and purified as described above. CXCL12 concentration was determined by direct amino acid analysis at the W. M. Keck facility (Yale University).

### Dose-Response of Fosfosal on CXCR4 Activation

Instead of a decrease in CXCL12 activity with any of the compounds, we observed an increase of activity with fosfosal. A dose-response experiment was used to determine the EC_50_ of fosfosal with 200 nM CXCL12 for activation of CXCR4 in *S. cerevisiae* with media described in the high throughput assay. Saturation could not be obtained with fosfosal but a non-linear fit was utilized to determine the EC_50_ using the Prism software [GraphPad, Inc., Log(agonist) vs. Response, Eq. 1]:

(1) $$y=\frac{B_{m\,i\,n}+\left(B_{m\,a\,x}-B_{m\,i\,n}\right)}{{\left(1+10^{L\,o\,g\,E\,C_{50}}-x\right)}^{*}\,H}$$

where *B*_min/max_ are the luminescence values at low and high concentrations of fosfosal, and *H* is the Hill Slope.

### Size Exclusion Chromatography With Multi-Angle Light Scattering (SEC-MALS)

An Agilent 960 HPLC system was used for analyzing CXCL12 with a Superdex 75 10/300 GL size exclusion column in tandem with Wyatt Dawn Helios II MALS/Wyatt T-rEx refractive index detectors (Wyatt Technology) under three different conditions: (A) 25 mM HEPES buffer at pH 7.4 with 500 mM NaCl, (B) same as (A) with 100 mM potassium phosphate, and (C) conditions as in (B) with 50 mM fosfosal.

### NMR Spectroscopy

Nuclear magnetic resonance experiments were carried out on Varian Inova and Bruker Avance NEO 600 MHz spectrometers. All spectra were collected at 25°C in 50 mM HEPES buffer at pH 7.4 or 50 mM MES buffer at pH 5.6. NMR spectra were processed with NMRPipe (Delaglio et al., 1995) and analyzed in SPARKY (Lee et al., 2015). Fosfosal, salicylate, or phosphate was titrated into CXCL12 until no additional shifts were observed in ^1^H^15^N NMR resonances. Chemical shift titrations for the determination of fosfosal affinity for CXCL12 were fit with Eq. 2 (adapted from a one-phase association model) and analyzed in Wolfram Alpha (Wolfram Research) and Prism:

(2) $$y=B_{u\,n\,s\,a\,t}+\left(B_{s\,a\,t}-B_{u\,n\,s\,a\,t}\right)\cdot \left(1-e\,x\,p^{\left(-K_{d}\cdot x\right)}\right)$$

where *B*_unsat_ and *B*_sat_ represent the boundaries (normalized chemical shift values) of free and fosfosal-bound CXCL12, respectively, and *K*_d_ is the dissociation constant.

## Results

### High-Throughput Screening Assay for Antagonists of CXCR4 Activity in *S. cerevisiae*

**FIGURE 1:**
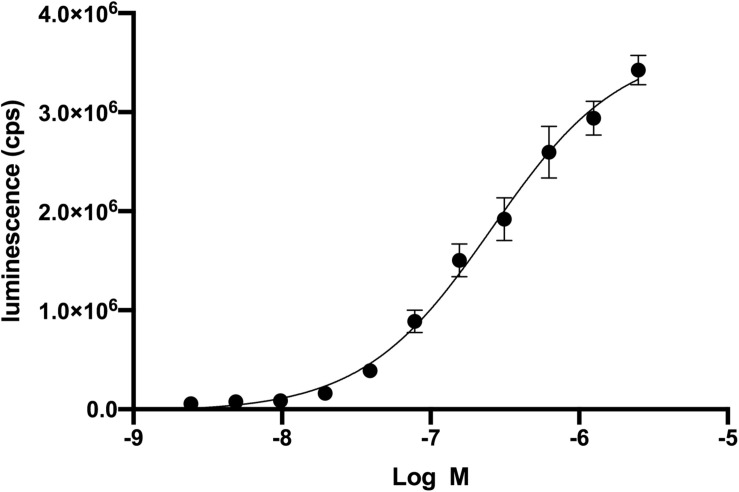
Recombinant CXCL12 activation of CXCR4 in *Saccharomyces cerevisiae*. Dose-response of CXCL12 activation of CXCR4 results in an EC_50_ of 269 nM. CXCR4 activation leads to the β-galactosidase enzymatic activity. Beta-glo (Promega) is a coupled enzyme reaction system to quantitate β-galactosidase by using a 6-O-β-galactopyranosyl-luciferin that is cleaved to luciferin and galactose. Luciferin is the substrate for the firefly luciferase and generates luminescence.

We used a genetic strain of *S. cerevisiae* expressing a functional human CXCR4 to optimize an assay to identify antagonists from a small library of bioactive molecules (Sachpatzidis et al., 2003). In a previous study, the cDNA for a library of ∼160,000 peptides, each with a signal sequence, was co-transfected into this genetically modified strain of *S. cerevisiae* (Supplementary Figure S1). More recently, we studied the mechanism of a CAM of CXCR4 and small molecules that bound to the CXCR4 CAM in the same strain of *S. cerevisiae* (Rosenberg et al., 2019). Neither of these previous studies required the addition of recombinant wild-type CXCL12, but in the current study an optimal concentration of CXCL12 was necessary to identify compounds that functionally decrease CXCL12-mediated activity of CXCR4. We first used CXCL12 in a dose-response experiment to determine an EC_50_ of 269 nM (Figure 1). The EC_50_ in mammalian cells is 7.9 nM (Murphy et al., 2007). The high EC_50_ relative to human cells reflects differences between *S. cerevisiae* and mammalian cells but did not prevent the optimization of the screen using variations of CXCL12 concentration, the number of *S. cerevisiae* cells, and time of incubation (Supplementary Figure S2). Of the 960 screened compounds, we were surprised to observe one, fosfosal, that increased CXCR4 activity by three standard deviation units above the average for all wells at about 20% above the control of 200 nM CXCL12 alone.

### Characterization and Mechanism of Fosfosal in CXCL12-Mediated CXCR4 Activation

Fosfosal is a salicylic acid derivative marketed in Spain as a NSAID (Rafanell et al., 1980). We evaluated its dose-response effects in the engineered *S. cerevisiae* strain and found that fosfosal is inactive when tested on its own (Supplementary Figure S3). Fosfosal only has activity in the presence of CXCL12 with an EC_50,app_ of 217 μM for activation of CXCR4 calculated through a non-linear fit (Equation 1), as fosfosal does not reach saturation due to its solubility (Supplementary Figure S3). We next explored whether there were any specific interactions of fosfosal with CXCL12 in either of its oligomeric states.

**FIGURE 2:**
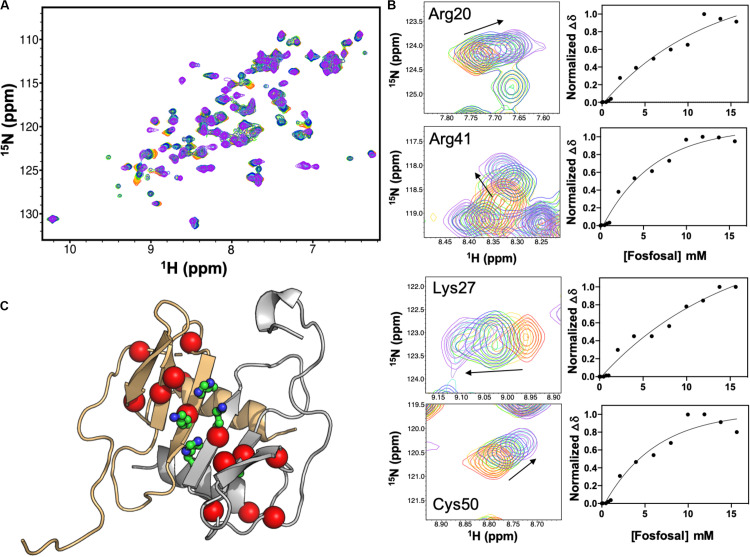
NMR titration of CXCL12 with fosfosal at pH 7.4. **(A)** Chemical shift perturbations in the ^1^H-^15^N HSQC NMR spectrum of 0.6 mM CXCL12 induced by 1–16 mM fosfosal (red → purple). **(B)** Selected resonances with significant chemical shift perturbations. Global fitting of eight chemical shift profiles gives an apparent binding affinity, *K*_d,app_ = 147 μM. **(C)** Structure of the CXCL12 dimer (PDB 4UAI) showing the monomeric units in gray and orange. Residues His25, Lys27, and Ala40, thought to be critical for dimerization (Gozansky et al., 2005), are shown in green sticks. Residues with significant chemical shift perturbations in fosfosal titrations are mapped onto the structure, clustering around the β-strand core and dimer interface.

Isotopically labeled ^15^N-CXCL12 was produced as described (Murphy et al., 2007). A global fit of eight CXCL12 NMR resonances with significant chemical shift perturbations upon titration of 16 mM fosfosal into CXCL12 yielded an apparent dissociation constant, *K*_d,app_ of 147 μM (Figures 2A,B) after accounting for the concentration of CXCL12 (0.6 mM). At this concentration, apo CXCL12 is expected to be dimeric. We mapped residues with significant chemical shift perturbations or line broadening at saturating fosfosal concentrations onto the CXCL12 structure (Figure 2C). Most notably, His25, Lys27, and Ala40 are in close proximity in the CXCL12 monomer and were proposed to be critical for CXCL12 dimerization in a prior study (Gozansky et al., 2005). Additional chemical shift perturbations localize to the area surrounding these residues spanning the β-strand core of the CXCL12 monomer-monomer interface (Figure 2C). Resonances selected for this binding analysis were in the fast exchange regime, although the addition of fosfosal modulates other sites of slow-to-intermediate exchange, most notably the N-terminus, upon titration into 0.6 mM CXCL12 (Supplementary Figures S4, S5). This suggests the interaction of fosfosal with CXCL12 may propagate a binding signal to the flexible N-terminal region, or alter its architecture.

**FIGURE 3:**
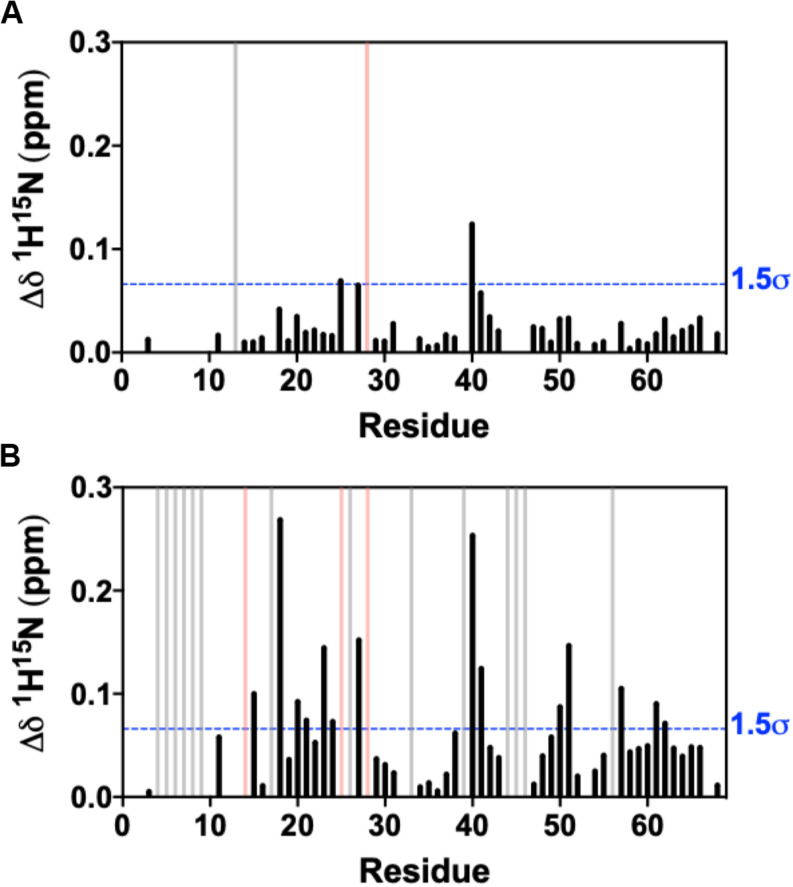
Combined ^1^H-^15^N NMR chemical shift perturbations from titration of 30 mM fosfosal stock solution into **(A)** 0.15 mM CXCL12 and **(B)** 0.6 mM CXCL12 at pH 7.4. Blue dashed lines indicate 1.5σ above the 10% trimmed mean of all shifts, gray bars denote peaks that became visible during the titration (intermediate → fast exchange), and pink bars represent peaks with observed line broadening.

We collected an additional ^1^H-^15^N NMR titration for fosfosal binding to 0.15 mM CXCL12, under buffer conditions and a concentration that favors CXCL12 monomers in solution. The ^1^H^15^N NMR spectra of CXCL12 at each concentration are very similar (Supplementary Figure S6), and Figure 3 shows chemical shift perturbations for fosfosal binding to CXCL12 under conditions favoring dimers and monomers. In the latter case, fosfosal does not induce chemical shift perturbations in 0.15 mM CXCL12 beyond 6 mM. The affected regions of the protein are consistent in both experiments (Figure 3), and the possibility that some contribution to the larger chemical shift perturbations in the dimer titration stem from structural effects propagated by the binding of a second fosfosal molecule to the adjacent CXCL12 subunit must be noted. Molecular docking of fosfosal with the CXCL12 monomer and dimer structures performed with HADDOCK (van Zundert et al., 2016), which incorporates NMR chemical shift data to define binding constraints, also indicates the interaction of fosfosal occurs with the CXCL12 monomer or dimer (Supplementary Figure S7). Interestingly, fosfosal does not modulate slow-to-intermediate exchange in NMR spectra of 0.15 mM CXCL12 (Figure 3 and Supplementary Figures S4, S5), as there are neither “satellite” peaks observed nor N-terminal resonances that reappear upon saturation with the ligand. This may be related to the ability of fosfosal to preferentially modulate dynamics in the CXCL12 dimer that in turn could alter the energetics of non-covalent interactions in the oligomer.

Size exclusion chromatography with multi-angle light scattering experiments show that the average molecular weight of CXCL12 in solution decreases from 11.6 to 9.4 kDa with 50 mM fosfosal (Supplementary Figure S8) suggesting that the equilibrium between monomer and dimer shifts toward the monomeric state in the presence of fosfosal. Given the SEC-MALS results in conjunction with the NMR data and computational modeling of fosfosal with both forms of CXCL12 using HADDOCK, we conclude fosfosal binds each monomer at surface residues at the dimer interface, affecting the monomer-monomer interactions, and increasing the concentration of the active monomer.

### Effects of Chemical Components of Fosfosal on CXCL12

**FIGURE 4:**
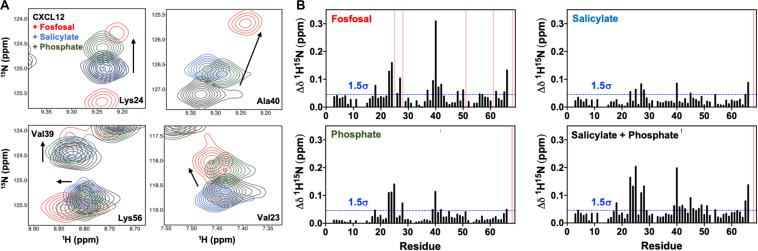
**(A)** Contribution of salicylate and phosphate moieties to fosfosal binding. Selected chemical shift perturbations induced by saturation of 0.6 mM CXCL12 (black) with fosfosal (red), sodium salicylate (blue), and phosphate (green). NMR spectra were collected in 50 mM MES buffer at pH 5.6, as acidic pH was previously shown to mitigate dimer formation (Veldkamp et al., 2005). **(B)** Combined ^1^H-^15^N NMR chemical shift perturbations observed for 0.6 mM CXCL12 with fosfosal (top left), sodium salicylate (top right), and phosphate (bottom left), and a plot of the perturbations observed from the additive effect of sodium salicylate and phosphate (bottom right). Blue dashed lines indicate 1.5σ above the 10% trimmed mean of all shifts and pink bars represent peaks with observed line broadening.

Fosfosal is composed of salicylate and phosphate moieties. In a previous study, negatively charged counter-ions such as phosphate, citrate, sulfate, the glycosaminoglycan heparin, or a non-acidic pH to keep the His25 side chain uncharged stabilized the dimer structure (Veldkamp et al., 2005). The heparin binding site was determined by both NMR (Veldkamp et al., 2005) and X-ray crystallography (Murphy et al., 2007). We studied the relative effects of the salicylate and phosphate moieties on CXCL12 for comparison to fosfosal. In order to mitigate effects from changes in the monomer–dimer equilibrium during these experiments, NMR chemical shifts were monitored upon saturation with fosfosal, sodium phosphate or sodium salicylate at pH 5.6 (Supplementary Figure S9), as acidic pH has been shown to favor monomeric CXCL12 even at high concentrations (van Zundert et al., 2016). The NMR spectrum of CXCL12 was fully assigned at pH 5.6 to account for some notable differences from the pH 7.4 spectrum caused by the change in pH (Supplementary Figure S10). The overall magnitude of the chemical shift perturbations of salicylate and phosphate is diminished relative to those caused by fosfosal, although they map to the same binding region of CXCL12 with residues in regions 24–27 and 39–42 most affected and statistically significant (Figure 4A). Together, the salicylate and phosphate moieties appear to have a predominantly additive effect, where the chemical shift profile of fosfosal is nearly recapitulated when its two components are combined (Figure 4B).

## Discussion

The largest protein family targeted by FDA-approved drugs is GPCRs, which play many physiological roles and are activated by photons, ions, small molecules, and proteins. The drugs that are approved for GPCRs are agonists, antagonists, inverse agonists, and modulators of activity. The dynamic nature of the GPCR protein family makes it amenable to identifying small molecules that bind to different conformational states. Over the past 15 years, technological advances have made the determination of three-dimensional structures of recombinant GPCRs possible for use in structure-guided drug design or virtual screening. Other methods to identify small molecule modulators rely on HTS followed by structure-activity relationships to identify. Optimized assays are capable of identifying small molecule compounds that function as agonists or antagonists. It is far more challenging to identify an agonist, which binds and induces conformational changes to activate a receptor than an antagonist that binds and blocks an agonist from binding to its receptor. Therefore, there are many antagonists for GPCRs but few agonists. For CXCR4, a small molecule agonist with moderate potency for CXCR4 is known (Mishra et al., 2016). Other strategies to modulate receptor activities are focused on chemokines instead of their receptors. A CXCL12 small molecule inhibitor is known to prevent activation of CXCR4 and CXCR7 at low micromolar levels (Veldkamp et al., 2010), but there are no known positive modulators of chemokines that increase the activity of their receptor.

The *S. cerevisiae* strain expressing a functional CXCR4 used for our HTS has both advantages and disadvantages. The advantages include ease of growth, nutrient selectivity for desired properties, simplicity of genetic modification, and the ability to couple G-protein to intracellular pathways resulting in a quantitative response. The expression of human CXCR4 in a non-mammalian cell also has disadvantages. For example, the EC_50_ for recombinant CXCL12 is ∼100-fold greater in *S. cerevisiae* relative to mammalian cells, likely due to a number of factors. Further, the cell wall of *S. cerevisiae* is a barrier for proteins under a variety of conditions. Another disadvantage is the inability to sulfate tyrosine residues, which contribute to chemokine affinity (Veldkamp et al., 2006). Tyrosine residues at positions 7, 12, and 21 in CXCR4 are not sulfated due to the absence of tyrosylprotein sulfotransferase-1 in the *S. cerevisiae* genome. There are also differences in the phospholipid bilayers of *S. cerevisiae* and mammalian cell membranes that may alter the conformational states and physiological function of any human GPCR. The ratio of specific phospholipids is different while other components may be entirely distinct. For example, cholesterol in mammalian cells is replaced by ergosterol in *S. cerevisiae*. CXCR4 and many other mammalian GPCRs bind cholesterol, which plays a role in stability and function (Liu et al., 2012; Pluhackova et al., 2016). None of these differences, however, prevented the development of a high-throughput assay for CXCR4.

We used a small library with many FDA-approved or bioactive drugs in a proof-of-principle study to determine whether *S. cerevisiae* can be optimized for an HTS for chemokine receptors. We identified fosfosal as a positive regulator of CXCR4, but only in the presence of CXCL12. The true EC_50_ of fosfosal could not be determined because it does not reach saturation (Supplementary Figure S3). Due to its weak affinity, we used orthogonal, *in vitro* techniques with purified recombinant protein to confirm binding and investigate its mechanism. The activity of fosfosal is due to its interactions with residues that map to surface residues of the CXCL12 dimer interface. Although CXCL12 dimers and higher order oligomers (Murphy et al., 2010) play an important role in chemokine function by forming a concentration gradient that is required for migration of cells (Proudfoot et al., 2003), it is the monomer that activates CXCR4 as previously shown by the three-dimensional structures of chemokine-receptor complexes (Burg et al., 2015; Qin et al., 2015; Zheng et al., 2017). The mechanism of how fosfosal increases the CXCL12-mediated CXCR4 activity is based on the following facts: (1) fosfosal increases the activity of CXCL12 in cellular studies with *S. cerevisiae*, (2) NMR suggests fosfosal interacts with both the dimer and monomer, saturating the monomer over a narrower concentration range of fosfosal, (3) fosfosal promotes a decrease in the average molecular weight of CXCL12 by SEC-MALS, and (4) the CXCL12 monomer is the active species. Molecular docking shows favorable interactions with the CXCL12 dimer, perhaps to modulate the monomer–dimer equilibrium via another conformational step to favor the monomeric active form. Fosfosal-induced conformational changes exclusive to the CXCL12 dimer would be consistent with NMR-detected changes in dynamics found only in this complex; however, we do not have crystallographic data that confirms the docked model. Additionally, the computations with HADDOCK are not precise enough to determine whether the monomer or dimer is favored *in vitro* or *in vivo*, as monomeric CXCL12 also strongly docks fosfosal. The majority of these data are consistent with a case where the interaction of fosfosal with monomeric CXCL12 is thermodynamically favored, which would be the expectation based on its activity. We also analyzed the contribution to binding of both the salicylate and phosphate chemical moieties of fosfosal, as both molecules form interactions with CXCL12 in docked complexes and display an additive effect in NMR chemical shift titrations. However, these smaller functional groups do not induce changes in the exchange regimes of amino acids observed with fosfosal (Supplementary Figures S4, S5).

We describe the first molecule that up-regulates CXCR4 activity and affects the monomer-dimer equilibrium of CXCL12 to favor the monomeric form. Since fosfosal is a weak up-regulator that was part of a 960-compound library, much larger compound libraries could be screened to identify more potent regulators of CXCL12. Alternatively, fosfosal analogs can be purchased or synthesized to test for increased the potency and induce CXCL12-mediated CXCR4 signaling. Known ligand databases (i.e., PubChem) have many fosfosal analogs that we will test with structure-based virtual and experimental screening in subsequent studies. This dimeric interaction interface can also be used to investigate molecules that stabilize the CXCL12 dimer and down-regulate CXCR4 activity. Finally, modification of this genetic strain of *S. cerevisiae* could be used for HTS of other chemokines, receptors, or GPCRs to identify tool compounds for studying these protein–receptor interactions.

## Data Availability Statement

The datasets generated for this study are available on request to the corresponding author.

## Author Contributions

JWM, DR, and JM were involved in the optimization of the high-throughput screening and carried out screening against the library. ES, CK, and JM were involved with expression, purification, and purification of labeled and unlabeled CXCL12, and carried out the NMR experiments. JWM was involved in studying fosfosal in *S. cerevisiae* and in carrying out SEC-MALS. All co-authors were involved in analyzing the data. GL and EL wrote the manuscript. All authors have given approval to the final version of the manuscript.

## Conflict of Interest

MH is now employed by Eli Lilly and Company. The remaining authors declare that the research was conducted in the absence of any commercial or financial relationships that could be construed as a potential conflict of interest.

## Abbreviations

CXCL12: CXC chemokine ligand 12
CXCR4: CXC chemokine receptor 2
fosfosal: 2-(phosphonooxy) benzoic acid
GPCR: G protein-coupled receptor.

## References

[B1] Aiuti A. Webb I. J. Bleul C. Springer T. Gutierrez-Ramos J. C. (1997). The chemokine SDF-1 is a chemoattractant for human CD34+ hematopoietic progenitor cells and provides a new mechanism to explain the mobilization of CD34+ progenitors to peripheral blood. *J. Exp. Med.* 185 111–120. 10.1084/jem.185.1.111 8996247 PMC2196104

[B2] Allen S. J. Crown S. E. Handel T. M. (2007). Chemokine: receptor structure, interactions, and antagonism. *Annu. Rev. Immunol.* 25 787–820. 17291188 10.1146/annurev.immunol.24.021605.090529

[B3] Bernhagen J. Krohn R. Lue H. Gregory J. L. Zernecke A. Koenen R. R. (2007). MIF is a noncognate ligand of CXC chemokine receptors in inflammatory and atherogenic cell recruitment. *Nat. Med.* 13 587–596. 10.1038/nm1567 17435771

[B4] Burg J. S. Ingram J. R. Venkatakrishnan A. J. Jude K. M. Dukkipati A. Feinberg E. N. (2015). Structural biology. Structural basis for chemokine recognition and activation of a viral G protein-coupled receptor. *Science* 347 1113–1117. 10.1126/science.aaa5026 25745166 PMC4445376

[B5] Chatterjee M. Borst O. Walker B. Fotinos A. Vogel S. Seizer P. (2014). Macrophage migration inhibitory factor limits activation-induced apoptosis of platelets via CXCR7-dependent Akt signaling. *Circ. Res.* 115 939–949. 10.1161/circresaha.115.305171 25266363

[B6] Crump M. P. Gong J. H. Loetscher P. Rajarathnam K. Amara A. Arenzana-Seisdedos F. (1997). Solution structure and basis for functional activity of stromal cell- derived factor-1, dissociation of CXCR4 activation from binding and inhibition of HIV-1. *EMBO J.* 16 6996–7007. 10.1093/emboj/16.23.6996 9384579 PMC1170303

[B7] Dale D. C. Bolyard A. A. Kelley M. L. Westrup E. C. Makaryan V. Aprikyan A. (2011). The CXCR4 antagonist plerixafor is a potential therapy for myelokathexis, WHIM syndrome. *Blood* 118 4963–4966. 10.1182/blood-2011-06-360586 21835955 PMC3673761

[B8] De Clercq E. (2009). The AMD3100 story: the path to the discovery of a stem cell mobilizer (Mozobil). *Biochem. Pharmacol.* 77 1655–1664. 10.1016/j.bcp.2008.12.014 19161986

[B9] Dealwis C. Fernandez E. J. Thompson D. A. Simon R. J. Siani M. A. Lolis E. (1998). Crystal structure of chemically synthesized [N33A] stromal cell-derived factor 1alpha, a potent ligand for the HIV-1 “fusin” coreceptor. *Proc. Natl. Acad. Sci. U.S.A.* 95 6941–6946. 10.1073/pnas.95.12.6941 9618518 PMC22694

[B10] Delaglio F. Grzesiek S. Vuister G. W. Zhu G. Pfeifer J. Bax A. (1995). NMRPipe: a multidimensional spectral processing system based on UNIX pipes. *J. Biomol. NMR* 6 277–293. 8520220 10.1007/BF00197809

[B11] Dohlman H. G. Song J. Ma D. Courchesne W. E. Thorner J. (1996). Sst2, a negative regulator of pheromone signaling in the yeast *Saccharomyces cerevisiae*: expression, localization, and genetic interaction and physical association with Gpa1 (the G-protein alpha subunit). *Mol. Cell. Biol.* 16 5194–5209. 10.1128/mcb.16.9.5194 8756677 PMC231520

[B12] Dong F. Harvey J. Finan A. Weber K. Agarwal U. Penn M. S. (2012). Myocardial CXCR4 expression is required for mesenchymal stem cell mediated repair following acute myocardial infarction. *Circulation* 126 314–324. 10.1161/circulationaha.111.082453 22685115

[B13] Donzella G. A. Schols D. Lin S. W. Este J. A. Nagashima K. A. Maddon P. J. (1998). AMD3100, a small molecule inhibitor of HIV-1 entry via the CXCR4 co- receptor. *Nat. Med.* 4 72–77. 10.1038/nm0198-072 9427609

[B14] Doranz B. J. Grovit-Ferbas K. Sharron M. P. Mao S. H. Goetz M. B. Daar E. S. (1997). A small-molecule inhibitor directed against the chemokine receptor CXCR4 prevents its use as an HIV-1 coreceptor. *J. Exp. Med.* 186 1395–1400. 10.1084/jem.186.8.1395 9334380 PMC2199097

[B15] Doring Y. Noels H. van der Vorst E. P. C. Neideck C. Egea V. Drechsler M. (2017). Vascular CXCR4 limits atherosclerosis by maintaining arterial integrity: evidence from mouse and human studies. *Circulation* 136 388–403. 10.1161/circulationaha.117.027646 28450349 PMC5777319

[B16] Doring Y. van der Vorst E. P. C. Duchene J. Jansen Y. Gencer S. Bidzhekov K. (2019). CXCL12 derived from endothelial cells promotes atherosclerosis to drive coronary artery disease. *Circulation* 139 1338–1340. 10.1161/circulationaha.118.037953 30865486 PMC6417827

[B17] Feng Y. Broder C. C. Kennedy P. E. Berger E. A. (1996). HIV-1 entry cofactor: functional cDNA cloning of a seven-transmembrane, G protein-coupled receptor. *Science* 272 872–877. 10.1126/science.272.5263.872 8629022

[B18] Fernandez E. J. Lolis E. (2002). Structure, function, and inhibition of chemokines. *Annu. Rev. Pharmacol. Toxicol.* 42 469–499. 11807180 10.1146/annurev.pharmtox.42.091901.115838

[B19] Gozansky E. Louis J. Caffrey M. Clore G. (2005). Mapping the Binding of the N-terminal Extracellular tail of the CXCR4 receptor to stromal cell-derived factor-1. *J. Mol. Biol.* 345 651–658. 10.1016/j.jmb.2004.11.003 15588815

[B20] Handel T. M. Johnson Z. Crown S. E. Lau E. K. Sweeney M. Proudfoot A. E. (2005). Regulation of protein function by glycosoaminoglycans–as exemplified by chemokines. *Annu. Rev. Biochem.* 74 385–410. 10.1146/annurev.biochem.72.121801.161747 15952892

[B21] Hendrix C. W. Collier A. C. Lederman M. M. Schols D. Pollard R. B. Brown S. (2004). Safety, pharmacokinetics, and antiviral activity of AMD3100, a selective CXCR4 receptor inhibitor, in HIV-1 infection. *J. Acqu. Immune Def. Syndrom.* 37 1253–1262. 10.1097/01.qai.0000137371.80695.ef 15385732

[B22] Hu X. Dai S. Wu W. J. Tan W. Zhu X. Mu J. (2007). Stromal cell derived factor-1 alpha confers protection against myocardial ischemia/reperfusion injury: role of the cardiac stromal cell derived factor-1 alpha CXCR4 axis. *Circulation* 116 654–663. 10.1161/circulationaha.106.672451 17646584 PMC3640445

[B23] Huang C. Gu H. Zhang W. Manukyan M. C. Shou W. Wang M. (2011). SDF-1/CXCR4 mediates acute protection of cardiac function through myocardial STAT3 signaling following global ischemia/reperfusion injury. *Am. J. Physiol. Heart Circ. Physiol.* 301 H1496–H1505. 21821779 10.1152/ajpheart.00365.2011 PMC3197365

[B24] Kofuku Y. Yoshiura C. Ueda T. Terasawa H. Hirai T. Tominaga S. (2009). Structural basis of the interaction between chemokine stromal cell-derived factor-1/CXCL12 and its G-protein-coupled receptor CXCR4. *J. Biol. Chem.* 284 35240–35250. 10.1074/jbc.m109.024851 19837984 PMC2787383

[B25] LaRocca T. J. Altman P. Jarrah A. A. Gordon R. Wang E. Hadri L. (2019). CXCR4 cardiac specific knockout mice develop a progressive cardiomyopathy. *Intern. J. Mol. Sci.* 20:2267. 10.3390/ijms20092267 31071921 PMC6539363

[B26] Lee W. Tonelli M. Markley J. L. (2015). NMRFAM-SPARKY: enhanced software for biomolecular NMR spectroscopy. *Bioinformatics* 31 1325–1327. 10.1093/bioinformatics/btu830 25505092 PMC4393527

[B27] Liu W. Chun E. Thompson A. A. Chubukov P. Xu F. Katritch V. (2012). Structural basis for allosteric regulation of GPCRs by sodium ions. *Science* 337 232–236. 10.1126/science.1219218 22798613 PMC3399762

[B28] McDermott D. H. Liu Q. Ulrick J. Kwatemaa N. Anaya-O’Brien S. Penzak S. R. (2011). The CXCR4 antagonist plerixafor corrects panleukopenia in patients with WHIM syndrome. *Blood* 118 4957–4962. 10.1182/blood-2011-07-368084 21890643 PMC3208300

[B29] McDermott D. H. Pastrana D. V. Calvo K. R. Pittaluga S. Velez D. Cho E. (2019). Plerixafor for the treatment of WHIM syndrome. *N. Engl. J. Med.* 380 163–170. 30625055 10.1056/NEJMoa1808575 PMC6425947

[B30] Mishra R. K. Shum A. K. Platanias L. C. Miller R. J. Schiltz G. E. (2016). Discovery and characterization of novel small-molecule CXCR4 receptor agonists and antagonists. *Sci. Rep.* 6:30155. 10.1038/srep30155 PMC4960487 27456816

[B31] Murphy J. W. Cho Y. Sachpatzidis A. Fan C. Hodsdon M. E. Lolis E. (2007). Structural and functional basis of CXCL12 (stromal cell-derived factor-1alpha) binding to heparin. *J. Biol. Chem.* 282 10018–10027. 10.1074/jbc.m608796200 17264079 PMC3684283

[B32] Murphy J. W. Yuan H. Kong Y. Xiong Y. Lolis E. J. (2010). Heterologous quaternary structure of CXCL12 and its relationship to the CC chemokine family. *Proteins* 78 1331–1337. 10.1002/prot.22666 20077567 PMC3021379

[B33] Peled A. Wald O. Burger J. (2012). Development of novel CXCR4-based therapeutics. *Expert Opin. Investig. Drugs* 21 341–353. 10.1517/13543784.2012.656197 22283809

[B34] Pluhackova K. Gahbauer S. Kranz F. Wassenaar T. A. Bockmann R. A. (2016). Dynamic cholesterol-conditioned dimerization of the G protein coupled chemokine receptor Type 4. *PLoS Comput. Biol.* 12:e1005169. 10.1371/journal.pcbi.1005169 27812115 PMC5094716

[B35] Proudfoot A. E. Handel T. M. Johnson Z. Lau E. K. LiWang P. Clark-Lewis I. (2003). Glycosaminoglycan binding and oligomerization are essential for the in vivo activity of certain chemokines. *Proc. Natl. Acad. Sci. U.S.A.* 100 1885–1890. 10.1073/pnas.0334864100 12571364 PMC149928

[B36] Qin L. Kufareva I. Holden L. G. Wang C. Zheng Y. Zhao C. (2015). Crystal structure of the chemokine receptor CXCR4 in complex with a viral chemokine. *Science* 347 1117–1122. 10.1126/science.1261064 25612609 PMC4362693

[B37] Rafanell J. G. Belles L. Sanchez M. S. Forn J. (1980). Pharmacological study of 2-phosphonoxybenzoic acid (fosfosal), a new analgesic drug. *Arzneimittelforschung* 30 1091–1098. 6251858

[B38] Rosenberg E. M. Harrison R. E. D. Tsou L. K. Drucker N. Humphries B. (2019). Functional characterization, dynamics, and mechanism of cxcr4 antagonists on a constitutively active mutant. *Cell Chem. Biol.* 26 662–673. 30827936 10.1016/j.chembiol.2019.01.012 PMC6736600

[B39] Sachpatzidis A. Benton B. K. Manfredi J. P. Wang H. Hamilton A. Dohlman H. G. (2003). Identification of allosteric peptide agonists of CXCR4. *J. Biol. Chem.* 278 896–907. 10.1074/jbc.m204667200 12417595

[B40] van Zundert G. C. P. Rodrigues J. Trellet M. Schmitz C. Kastritis P. L. Karaca E. (2016). The HADDOCK2.2 web server: user-friendly integrative modeling of biomolecular complexes. *J. Mol. Biol.* 428 720–725. 10.1016/j.jmb.2015.09.014 26410586

[B41] Veldkamp C. T. Peterson F. C. Pelzek A. J. Volkman B. F. (2005). The monomer-dimer equilibrium of stromal cell-derived factor-1 (CXCL 12) is altered by pH, phosphate, sulfate, and heparin. *Protein Sci.* 14 1071–1081. 10.1110/ps.041219505 15741341 PMC2253449

[B42] Veldkamp C. T. Seibert C. Peterson F. C. Sakmar T. P. Volkman B. F. (2006). Recognition of a CXCR4 sulfotyrosine by the chemokine stromal cell-derived factor-1[alpha] (SDF-1[alpha]/CXCL12). *J. Mol. Biol.* 359 1400–1409. 10.1016/j.jmb.2006.04.052 16725153 PMC2670582

[B43] Veldkamp C. T. Ziarek J. J. Peterson F. C. Chen Y. Volkman B. F. (2010). Targeting SDF-1/CXCL12 with a ligand that prevents activation of CXCR4 through structure-based drug design. *J. Am. Chem. Soc.* 132 7242–7243. 10.1021/ja1002263 20459090 PMC2941798

[B44] Walters K. B. Green J. M. Surfus J. C. Yoo S. K. Huttenlocher A. (2010). Live imaging of neutrophil motility in a zebrafish model of WHIM syndrome. *Blood* 116 2803–2811. 10.1182/blood-2010-03-276972 20592249 PMC2974588

[B45] Zernecke A. Bot I. Djalali-Talab Y. Shagdarsuren E. Bidzhekov K. Meiler S. (2008). Protective role of CXC receptor 4/CXC ligand 12 unveils the importance of neutrophils in atherosclerosis. *Circ. Res.* 102 209–217. 10.1161/circresaha.107.160697 17991882

[B46] Zhang J.-H. Chung T. D. Y. Oldenburg K. R. (1999). A simple statistical parameter for use in evaluation and validation of high throughput screening assays. *J. Biomol. Screen.* 4 67–73. 10.1177/108705719900400206 10838414

[B47] Zhang W. B. Navenot J. M. Haribabu B. Tamamura H. Hiramatu K. Omagari A. (2002). A point mutation that confers constitutive activity to CXCR4 reveals that T140 is an inverse agonist and that AMD3100 and ALX40-4C are weak partial agonists. *J. Biol. Chem.* 277 24515–24521. 10.1074/jbc.m200889200 11923301

[B48] Zheng Y. Han G. W. Abagyan R. Wu B. Stevens R. C. Cherezov V. (2017). Structure of CC chemokine receptor 5 with a potent chemokine antagonist reveals mechanisms of chemokine recognition and molecular mimicry by HIV. *Immunity* 46 1005–1017. 28636951 10.1016/j.immuni.2017.05.002 PMC5572563

