# Supplementary material for: High-Throughput Screening of a Functional Human CXCL12-CXCR4 Signaling Axis in a Genetically Modified S. cerevisiae: Discovery of a Novel Up-Regulator of CXCR4 Activity
Source: Front Mol Biosci. 2020 Jul 16;7:164. doi: 10.3389/fmolb.2020.00164 (PMC7378375; doi:10.3389/fmolb.2020.00164)
Supplement: Supplementary file 1 [file Image_1.pdf]

Supporting information

High-throughput screening of a functional human CXCL12-CXCR4 in a genetically modified *S. cerevisiae*: discovery of a novel up-regulator of CXCR4 activity

James W. Murphy[‡], Deepa Rajasekaran[‡], Janie Merkel[§], Erin Skeens[‖], Michael Hodsdon[#,⊥,†],

George P. Lisi[‖], and Elias Lolis*[‡,⊥]

Departments of [‡]Pharmacology, [§]Yale Center for Molecular Discovery, [#]Laboratory Medicine, Yale University School of Medicine, New Haven, CT, [⊥]Yale Cancer Center, New Haven, CT, [‖]Department of Molecular Biology, Cell Biology & Biochemistry, Brown University, Providence, RI

***Keywords:* Chemokine, G protein-coupled receptor, CXCL12, CXCR4, high-throughput screening, fosfosal, NMR**

Correspondence: elias.lolis@yale.edu

Content

Figure S1 – Schematic of genetically modified *S. cerevisiae*
Figure S2 – Final optimization of ligand screen parameters
Figure S3 – Effect of fosfosal on CXCR4-expressing *S. cerevisiae*
Figure S4 – Fosfosal-induced intermediate-to-fast exchange in CXCL12
Figure S5 – Fosfosal-induced slow exchange in CXCL12
Figure S6 – NMR spectra of 0.15 mM and 0.60 mM apo CXCL12
Figure S7 – Molecular docking of fosfosal to monomeric and dimeric CXCL12 via HADDOCK2.4
Figure S8 – SEC-MALS of CXCL12
Figure S9 – NMR spectra of CXCL12 in the presence of fosfosal, salicylate, and phosphate
Figure S10 – NMR spectra of apo CXCL12 at pH 5.6 and pH 7.4

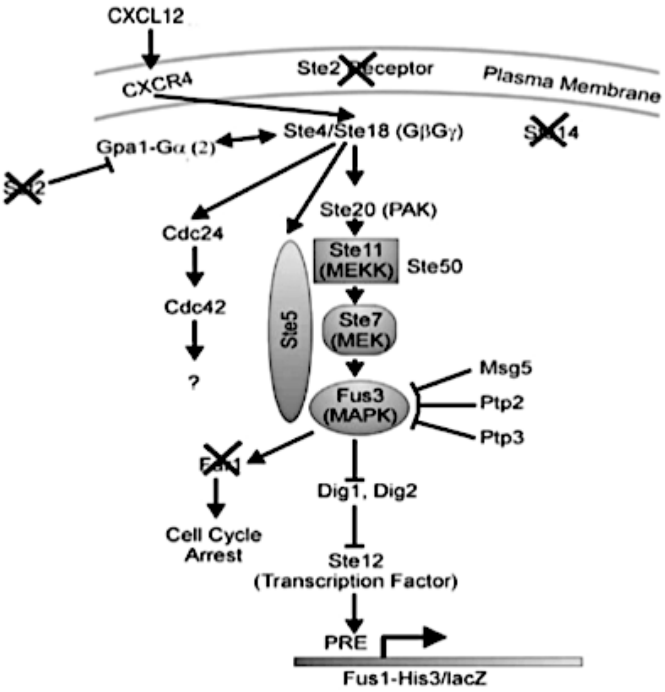

Figure S1. Genetic changes in *S. cerevisiae* that results in CXCL12 to activate CXCR4, induce the MAP kinase signaling pathway, and resulting in the expression of the *lacZ* gene. This figure is originally found in one of our previous studies of CXCR4 in *S. cerevisiae* (Sachpatzidis, *et al*, **2003**. *J. Biol. Chem. 278*, 896-907).

A

Cells/well	No CXCL12 Mean Lumin.	Std. Dev.	CV	+ CXCL12 Mean Lumin.	Std. Dev.	CV	+ CXCL12 & AMD3100 Mean Lumin.	Std. Dev.	CV
220000	7692	590	8%	89170	11167	8%	18025	3437	8%
110000	6691	786	12%	237484	29570	12%	30203	11760	12%
55000	6341	952	15%	303694	21226	7%	28208	7332	26%
27500	5433	846	16%	269194	26915	10%	11019	4543	41%
13750	4874	740	15%	219217	23424	11%	9724	2535	26%
6875	3752	688	18%	135452	17489	13%	5765	1691	29%
3437	3082	283	9%	82747	10696	13%	3509	419	12%
1718	2799	305	11%	36840	12795	35%	2785	130	5%

B

Cells/well	S/N CXCL12/No CXCL12	CXCL12/No CXCL12
220000	12	5
110000	35	8
55000	48	11
27500	50	24
13750	45	23
6875	36	23
3437	27	24
1718	13	13

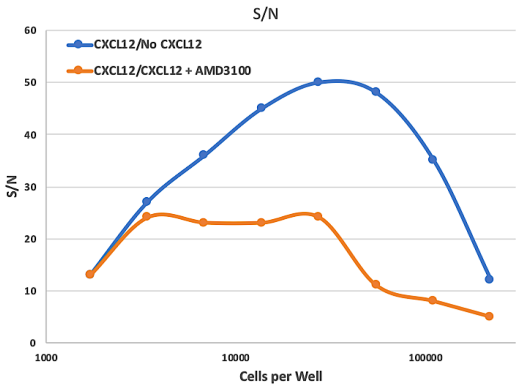

C

Cells/well	Z' CXCL12/No CXCL12	CXCL12/No CXCL12
220000	0.57	0.38
110000	0.61	0.4
55000	0.78	0.69
27500	0.68	0.63
13750	0.66	0.63
6875	0.59	0.56
3437	0.59	0.58
1718	-0.15	-0.14

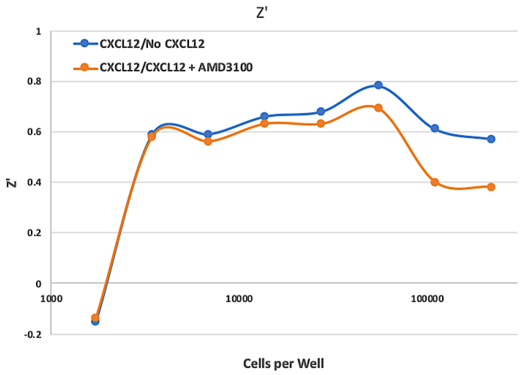

Figure S2. Final optimization of CXCL12 assay for agonist/antagonists screens. **(A)** Summary of experimental luminescence values (mean ± std. dev., n=8) determined as a function of cells per well (20 μL final volume) in the absence of CXCL12 (left), the presence of CXCL12 (200 nM, center) and the presence of CXCL12 (200 nM) and the known antagonist AMD3100 (10 μM, right). Coefficients of variation (CV) are also reported. **(B)** Experimental signal-to-noise (S/N) and **(C)** Z'-scores for the data collected in the presence of CXCL12 in reference to the control (absence of CXCL12, blue) and CXCL12 + AMD3100 (orange). Z' scores were calculated as $Z' = 1 - \frac{3\,x\,(\sigma_p - \sigma_n)}{|\mu_p - \mu_n|}$, where σ is the standard deviation and μ is the mean of the data.

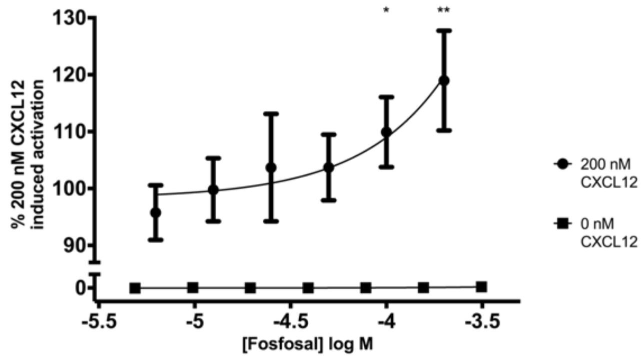

Figure S3. Effect of fosfosal on CXCR4-expressing *S. cerevisiae*. Dose-response fosfosal-induced CXCR4 activation in the presence and absence of 200 nM CXCL12. Only in the presence of CXCL12 is fosfosal active. Using 200 nM CXCL12, the dose-response of fosfosal results in an EC_{50} of 217 μM based on the β-galactosidase-mediate luciferase assay using β-glo. Statistics were calculated as unpaired t-test with Welch's correction and values were two-tailed: *, p value ≤ 0.05; **, p value ≤ 0.01

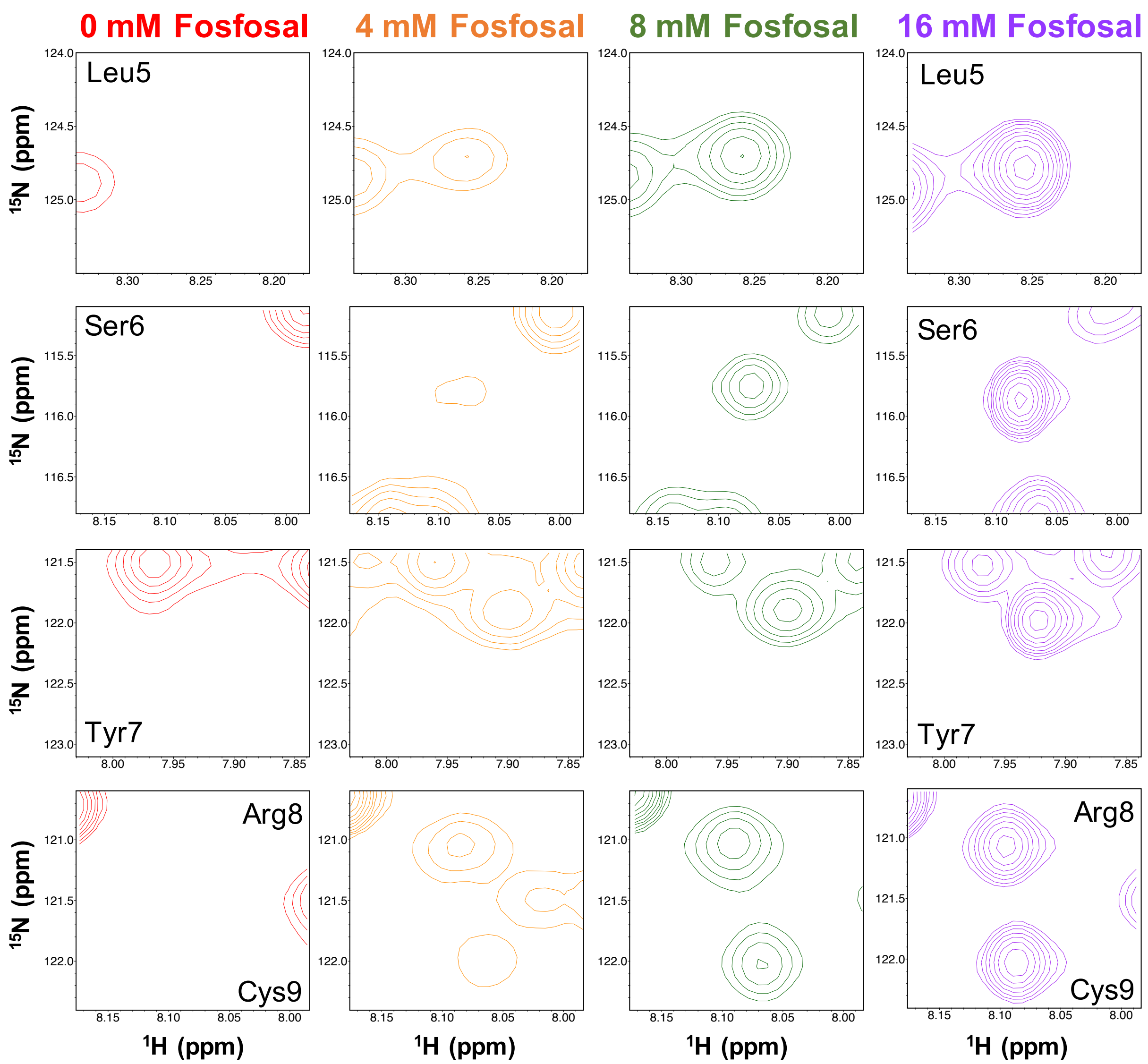

Figure S4. Alterations of intermediate-to-fast exchange of the CXCL12 N-terminus by fosfosal. Selected resonances from $^{1}H^{15}N$ HSQC spectra of 0.6 mM CXCL12 with 0 mM fosfosal (red), 4 mM fosfosal (orange), 8 mM fosfosal (green), and 16 mM fosfosal (purple) at pH 7.4 demonstrate that N-terminal resonances become visible over the course of the titration, suggesting fosfosal propagates a binding signal to this region of the protein. As shown in **Fig. 3**, this effect is not observed in identical fosfosal titrations carried out on 0.15 mM CXCL12, indicating a process specific to the dimer structure.

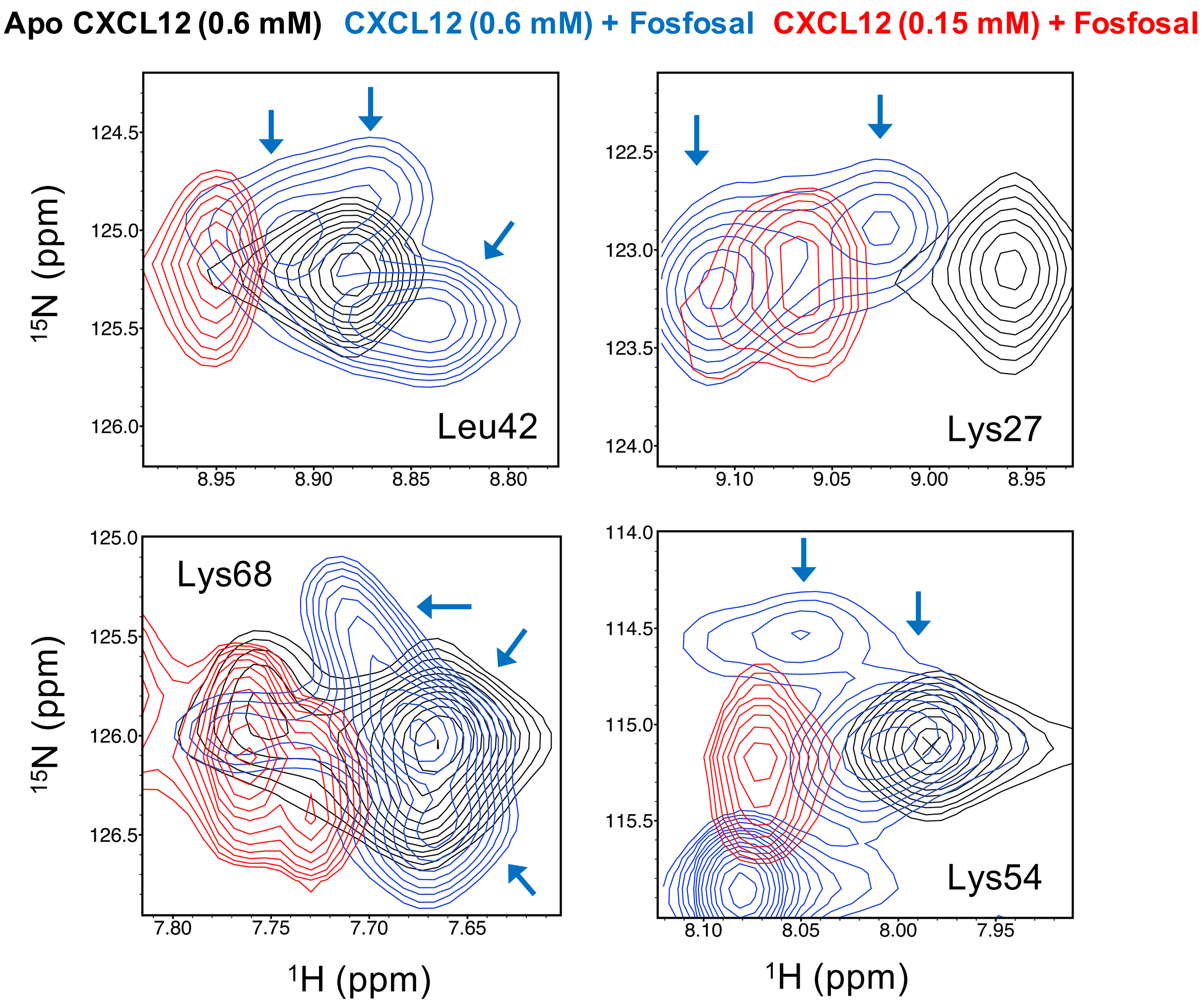

Figure S5. Fosfosal-induced slow exchange observed at high concentrations of CXCL12. $^{1}H^{15}N$ HSQC spectral overlays of selected resonances from spectra of 0.6 mM apo CXCL12 (black), 0.6 mM CXCL12 with fosfosal (blue) and 0.15 mM CXCL12 with fosfosal (red). Sites of slow exchange indicated with blue arrows are observed in the dimeric concentration regime of CXCL12 (0.6 mM) saturated with fosfosal, but are not observed at a high concentrations of apo CXCL12 or at low concentrations of CXCL12 (0.15 mM) saturated with fosfosal.

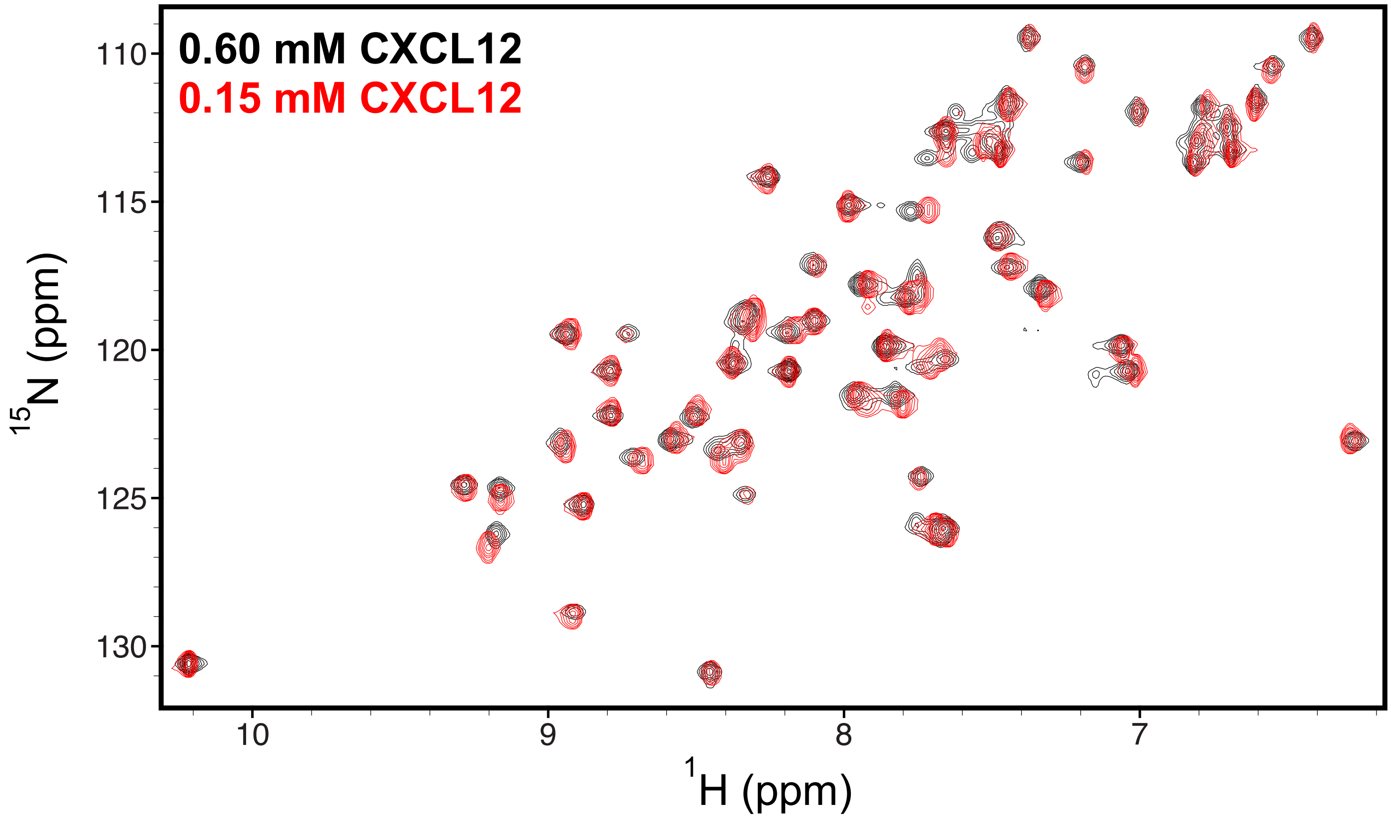

Figure S6: $^1H^{15}N$ HSQC spectral overlay of 0.6 mM apo CXCL12 (black) and 0.15 mM apo CXCL12 (red).

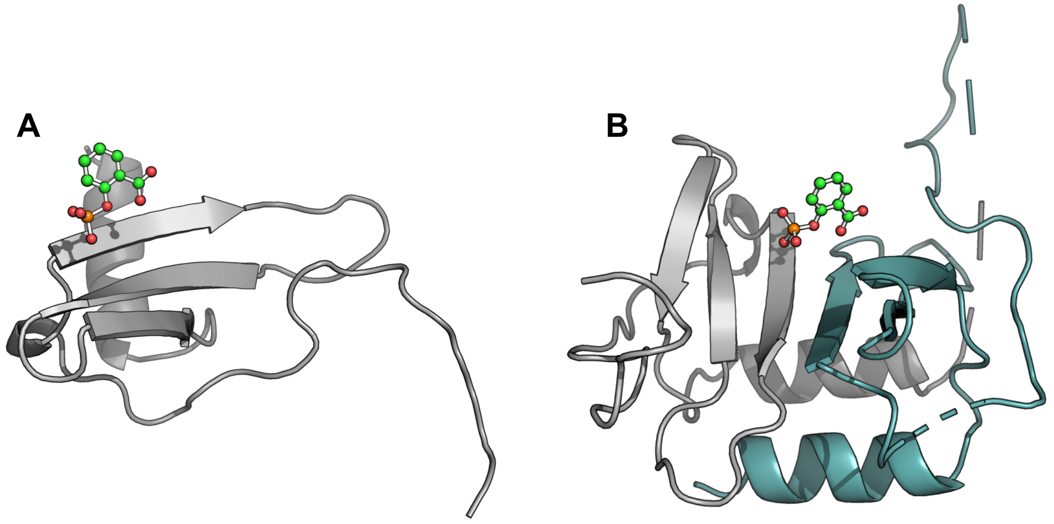

Figure S7. Molecular docking of fosfosal to CXCL12 shows an interaction at the β-strand core and dimer interface. HADDOCK2.4 (Zundert, et al *J. Mol. Biol.* **2016**, *428*, 720-725) was utilized for the docking of fosfosal (CID: 3418) to (A) the CXCL12 monomer (PDB: 2KEC) and (B) the CXCL12 dimer (PDB: 4UAI). Residues with significant chemical shift perturbations identified in the NMR titration of fosfosal into 0.6 mM CXCL12 at pH 7.4 (**Fig. 2**) were used to define Ambiguous Interaction Restraints (AIRs). Those residues, including Val18, Val23, Lys27, Ala40, Arg41, Ile51, Trp57, were set as "active" residues and HADDOCK2.4 automatically generated "passive" residues based on solvent accessibility and proximity to the active residues within a 6.5 Å radius. HADDOCK2.4 default parameters were used for the remainder of the docking simulations. HADDOCK2.4 identifies clusters of docking structures based on similar HADDOCK scores, a weighted sum of various calculated energy parameters. The structures presented in (A) and (B) have the lowest HADDOCK scores, which represent the most energetically favorable docking structures for each complex. The HADDOCK score for the CXCL12 monomer is -63.5 +/- 2.7 and for the dimer is -91.1 +/- 2.4, indicating that the docking of fosfosal to the CXCL12 dimer is more energetically favorable than to the CXCL12 monomer. The use of the HADDOCK webserver, as well as the determination of the default parameters used in the docking studies, are described in: van Zundert, Rodrigues, Trellet, Schmitz, Kastritis, Karaca, Melquiond, van Dijk, de Vries and Bonvin (2016). "The HADDOCK2.2 webserver: User-friendly integrative modeling of biomolecular complexes." *J. Mol. Biol.*, **428**, 720-725.

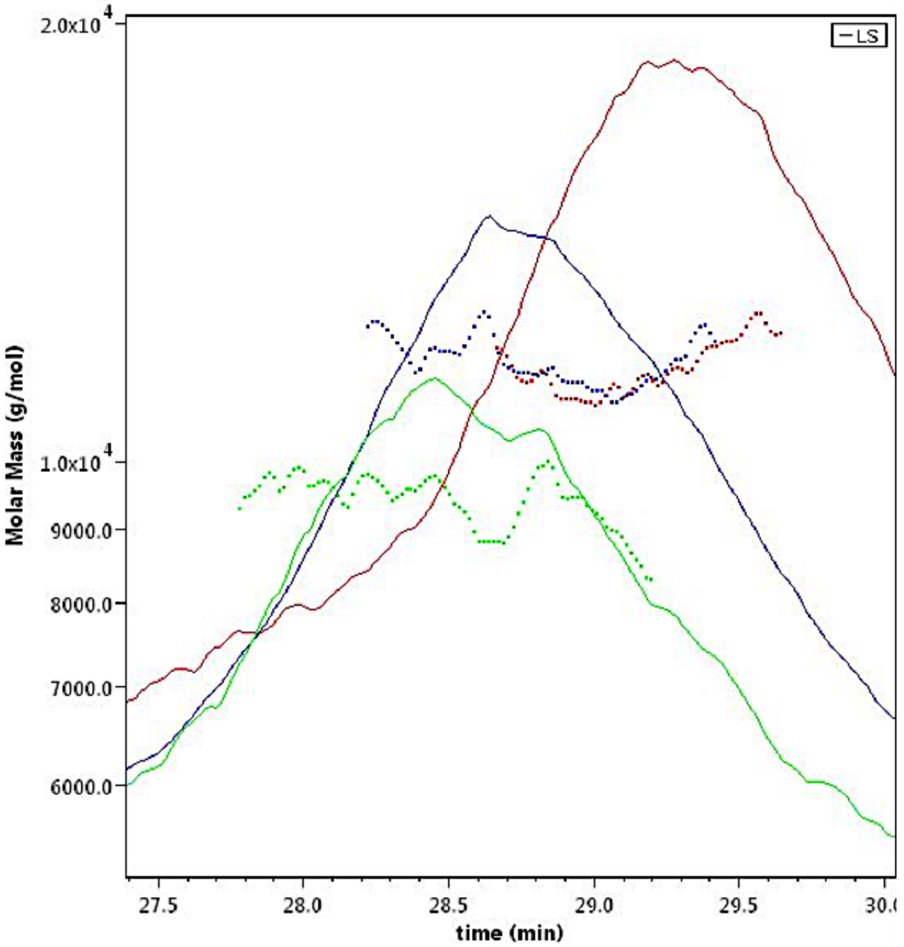

Figure S8. Size exclusion chromatography multi-angle light scattering of CXCL12 in 50 mM Hepes, pH 7.4, and 500 mM NaCl (red), CXCL12 in 50 mM Hepes, pH 7.4, 100 mM sodium phosphate, and 500 mM NaCl (purple), and CXCL12 in 50 mM Hepes, pH 7.4, 100 mM sodium phosphate, 500 mM NaCl, and 50 mM fosfosal (green). The dotted lines are the molecular weights and the curves are the results of light scattering. The molecular weight of CXCL12 with fosfosal (and phosphate) is 9.4 kDa versus 11.7 kDa and 11.6 kDa with and without phosphate, respectively. The molecular weight, based on the amino acid sequence of CXCL12 used in these experiments, is 7.98 kDa.

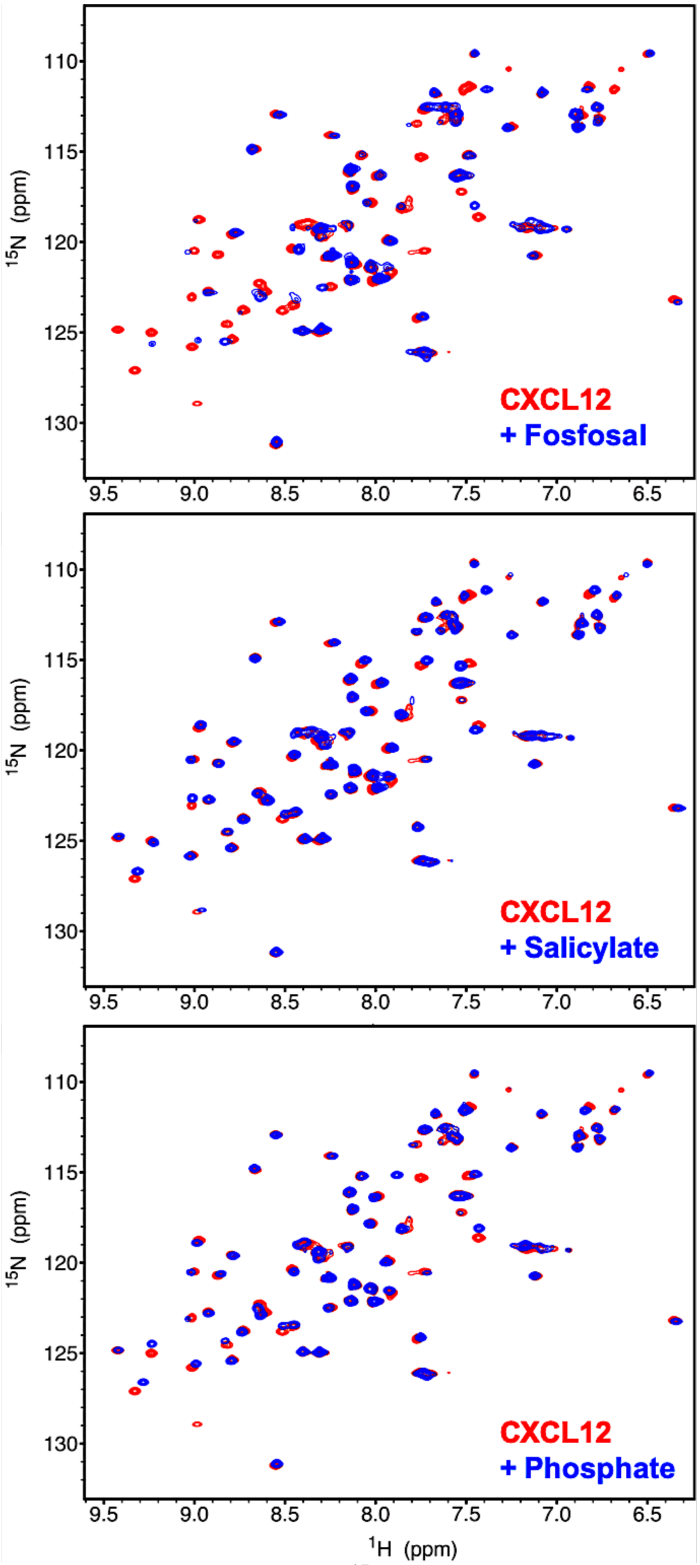

Figure S9. Solution NMR spectra of 0.6 mM ^{15}N-CXCL12 (red) overlaid after saturation with fosfosal (top), salicylate (middle), or phosphate (bottom) at pH 5.6. Ligand-bound spectra are shown in blue.

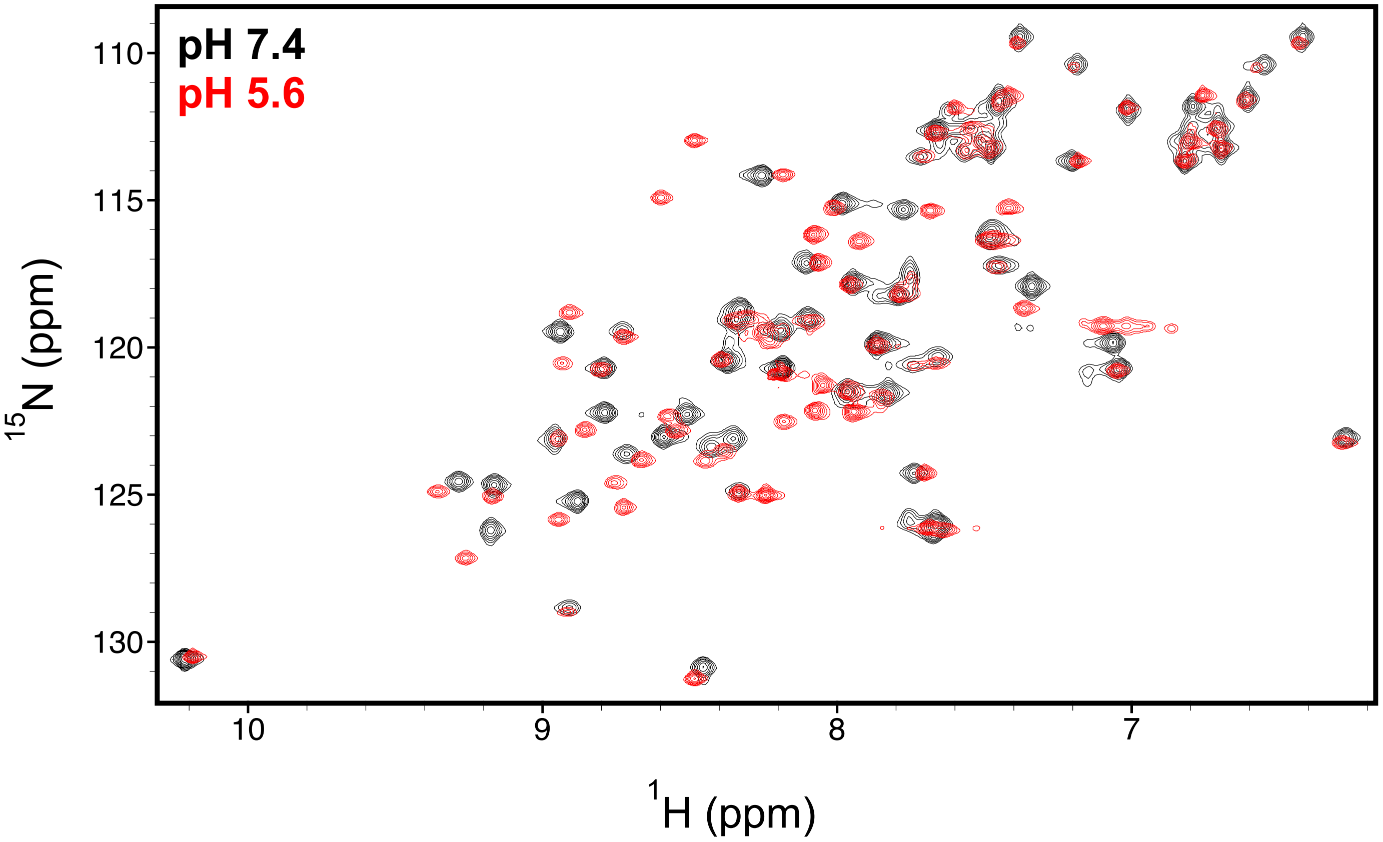

Figure S10. $^{1}H^{15}N$ HSQC spectral overlay of 0.6 mM apo CXCL12 at pH 7.4 (black) and pH 5.6 (red). Resonances shown in red without a corresponding black resonance are those belonging to the N-terminal amino acids of CXCL12.
